# Sulfatide decreases the resistance to stress-induced apoptosis and increases P-selectin-mediated adhesion: a two-edged sword in breast cancer progression

**DOI:** 10.1186/s13058-018-1058-z

**Published:** 2018-11-06

**Authors:** Jaroslaw Suchanski, Jedrzej Grzegrzolka, Tomasz Owczarek, Pawel Pasikowski, Aleksandra Piotrowska, Bartlomiej Kocbach, Aleksandra Nowak, Piotr Dziegiel, Andrzej Wojnar, Maciej Ugorski

**Affiliations:** 1 Department of Biochemistry and Molecular Biology, Faculty of Veterinary Medicine, Wroclaw University of Environmental and Life Sciences, C.K. Norwida 31, 50-375 Wroclaw, Poland; 2 Department of Histology and Embryology, Wroclaw Medical University, Wroclaw, Poland; 3 EIT+ Wroclaw Research Center, Wroclaw, Poland; 4 Laboratory of Glycobiology, Hirszfeld Institute of Immunology and Experimental Therapy, Polish Academy of Sciences, Wroclaw, Poland; 5 Department of Physiotherapy, Wroclaw University School of Physical Education, Wroclaw, Poland; 6 Lower Silesian Oncology Center, Wroclaw, Poland; 7 Present address: Department of Medicine, Herbert Irving Comprehensive Cancer Center, Columbia University Medical Center, New York, NY USA

**Keywords:** Breast cancer, Sulfatide, Galactosyloceramide, Apoptosis, Platelets, P-selectin, Adhesion

## Abstract

**Background:**

We have previously shown that galactosylceramide (GalCer) affects the tumourigenic and metastatic properties of breast cancer cells by acting as an anti-apoptotic molecule. Since GalCer is a precursor molecule in the synthesis of sulfatides, the present study was aimed to define the role of sulfatides in apoptosis and breast cancer progression.

**Methods:**

Expression of GAL3ST1 in breast cancer cell lines and breast cancer tissue specimens was analysed using real-time PCR, western blotting and immunohistochemistry analysis. The amount of sulfatide, GalCer and ceramide was analysed by thin-layer chromatography binding assay and by the modified hydrophilic interaction liquid chromatography coupled with electrospray mass spectrometry methodology. The tumourigenicity of cancer cells was analysed by an in-vivo tumour growth assay. Apoptotic cells were detected based on caspase-3 activation and the TUNEL assay. The interaction of breast cancer cells with P-selectin or E-selectin was analysed using the flow adhesion assay. The ability of sulfatide-expressing cells to activate and aggregate platelets was studied using the flow-cytometry-based aggregation assay.

**Results:**

Using two models of breast cancer, T47D cells with blocked synthesis of sulfatide and MDA-MB-231 cells with neosynthesis of this glycosphingolipid, we showed that high sulfatide levels resulted in increased sensitivity of cancer cells to apoptosis induced by hypoxia and doxorubicin in vitro, and decreased their tumourigenicity after transplantation into athymic nu/nu mice. Accordingly, a clinical study on GAL3ST1 expression in invasive ductal carcinoma revealed that its elevated level is associated with better prognosis. Using MDA-MB-231 cells with neosynthesis of sulfatide we also showed that sulfatide is responsible for adhesion of breast cancer cells to P-selectin-expressing cells, including platelets. Sulfatide also acted as an activating molecule, increasing the expression of P-selectin.

**Conclusions:**

This study demonstrates that increased synthesis of sulfatide sensitises cancer cells to microenvironmental stress factors such as hypoxia and anticancer drugs such as doxorubicin. However, sulfatide is probably not directly involved in apoptotic cascades, because its increased synthesis by GAL3ST1 decreased the amounts of its precursor, GalCer, a known anti-apoptotic molecule. On the other hand, our data support the view that sulfatides are malignancy-related adhesive molecules involved in activating and binding P-selectin-expressing platelets to breast cancer cells.

**Electronic supplementary material:**

The online version of this article (10.1186/s13058-018-1058-z) contains supplementary material, which is available to authorized users.

## Background

Sulfatides (SM4) are sulfated galactosylceramides (GalCer) present in the external leaflet of the plasma membrane in a variety of mammalian cells. Increased amounts of sulfatides have been found in colon, ovarian and gastric cancers [1–4], as well as renal cell and hepatocellular carcinomas [5, 6]. Differential expression of sulfatides was observed in lung carcinoma, with higher levels in adenocarcinomas than in squamous cell carcinoma and undifferentiated small cell carcinoma [7]. In colorectal and ovarian carcinoma, an increase in sulfatides correlated with poor prognosis [8, 9]. However, little is known about the biological role of sulfatides in cancer progression. It has been proposed, but not experimentally proven, that sulfatides present on the surface of cancer cells are ligands for P-selectin expressed by activated endothelial cells, and such interactions facilitate the formation of aggregates, which in turn increase their metastatic potential [10]. More recently, sulfated galactocerebroside was described as a P-selectin ligand on MC-38 murine colon carcinoma cells [11]. These studies revealed that in-vitro adhesion of activated platelets expressing P-selectin to MC-38 cells is mediated solely through sulfatides present on the latter. Such interactions were also observed in vivo. When mice were transplanted intravenously with MC-38 cells, their aggregates with platelets were observed in the lungs after 30 min.

It has been shown that SM4 is produced by several breast cancer cell lines [12]. In mammalian cells, sulfatides are synthesised by highly specific, late Golgi apparatus-localised 3′-phosphoadenosine-5′-phosphosulfate galactosylceramide sulfotransferase (GAL3ST1) (cerebroside sulfotransferase (CST); EC 2.8.2.11), which is responsible for 3-O-sulfation of galactose residue in GalCer molecules [13]. We recently showed that GalCer affects the tumourigenic and metastatic properties of breast cancer cells and acts as an anti-apoptotic molecule [14]. Since it was proposed that the balance between specific sphingolipid species is a critical rheostat for regulation of cellular apoptosis [15], the present study was undertaken to define the role of sulfatides in apoptosis and breast cancer progression. We show for the first time that sulfatides act not only as adhesive molecules, but are also involved in programmed cell death together with GalCer [14]. This highlights the importance of the glycolipids as regulators of cancer progression.

## Methods

### Breast cancer tissue specimens

The study was carried out on archival paraffin blocks of 232 randomly selected breast cancer patients (mean age 58.25 years) diagnosed between 2004 and 2009 at the Lower Silesian Oncology Centre in Wroclaw, Poland. All patients were treated by mastectomy, quadrantectomy and/or axillary lymph node resection. Of the patients, 94.4% (219 patients) and 85.34% (198 patients) were treated by adjuvant chemotherapy and hormonotherapy, respectively. All HER2-positive patients (14.66%, 34 patients) were additionally subjected to immunotherapy. The patients’ clinical and pathological data (presented in Table 1) were obtained from the hospital archives. Tumour histological type and malignancy grade (G) were determined according to World Health Organization (WHO) criteria [16]. The patients were followed for 121.41 months (range 1–161 months) and the median follow-up was 141.9 months.

**Table 1:** Clinical and pathological characteristics of breast cancer cases

Parameter	Patients		GAL3ST1		
	IHC (*N* = 232)	%	IHC negative	IHC positive	*p* value (chi-square^a^ or Fisher exact test^b^)
Age					
≤ 50 years	56	24.14	25	31	0.0904^b^
> 50 years	176	75.86	102	74	
Tumour grade					
G1	67	28.88	30	38	**0.0215** ^**b**^
G2	96	41.38	53	42	
G3	55	23.71	38	17	
No data	14	6.03			
Tumour size					
pT1	119	51.29	66	53	0.8706^b^
pT2	75	32.33	45	31	
pT3	4	1.72	2	2	
pT4	22	9.48	11	11	
	14	6.03			
Lymph nodes					
pN0	87	37.50	53	34	0.4386^b^
pN1–pN3	67	28.88	46	30	
pNx		0.00			
No data	12	5.17			
Stage					
I	53	22.84	33	20	0.6406^a^
II	60	25.86	35	25	
III	41	17.67	24	17	
IV	1	0.43	0	1	
No data	78	33.62			
Oestrogen receptor					
Negative	52	22.41	36	16	**0.0203** ^**b**^
Positive	167	71.98	85	82	
No data	13	5.60			
Progesterone receptor					
Negative	69	29.74	40	28	0.4503^b^
Positive	150	64.66	80	70	
No data	13	5.60			
HER2					
Negative	188	81.03	97	90	0.0894^b^
Positive	34	14.66	23	11	
No data	10	4.31			
Molecular tumour types					
Triple negative	18	7.6	11	7	0.5492^b^
Other types	199	85.78	107	92	
No data	10	4.31			
Chemotherapy					
Negative	13	5.60	8	5	0.7764^b^
Positive	219	94.4	119	100	
Hormonal therapy					
Negative	32	13.79	20	12	0.3398^b^
Positive	198	85.34	103	95	
No data	2	0.8			

Bold values (*p* < 0.05) are statistically significant

*HER2* human epidermal growth factor receptor 2, *IHC* immunohistochemistry, *Gal3ST1* galactosylceramide sulfotransferase

### Cell lines

The breast cancer cell lines MCF7, SKBR3 and BT-474 were obtained from the Cell Line Collection of the Hirszfeld Institute of Immunology and Experimental Therapy (Wroclaw, Poland). T47D and MDA-MB-231 breast cancer cell lines as well as CHO-Pro5 cells were obtained from the American Type Culture Collection (Manassas, VA, USA). CHO-Pro5 cells that expressed human E-selectin have been described elsewhere [17]. All human cell lines were authenticated by the ATCC Cell Line Authentication Service using Short Tandem Repeat analysis. The cells were cultured in α-minimum essential medium (αMEM) supplemented with 10% fetal calf serum (FCS; Cytogen, Lodz, Poland), 2 mM l-glutamine and antibiotics.

### In-vivo tumour growth assay

The animal study was approved by the Second Local Ethic Committee for Animal Experimentation (Wroclaw, Poland). Six-week-old athymic nude Crl:NU(Ncr)-Foxn1nu female mice were purchased from Charles River Laboratories (Sulzfeld, Germany) and kept under specific pathogen-free conditions at room temperature (RT). Human breast cancer cells were harvested by trypsinisation, washed with PBS and resuspended in the same buffer. Cell suspensions (2 × 10^6^ cells/100 μl PBS) were mixed with the same volume of ice-cold BD Matrigel Matrix High Concentration (Becton Dickinson, San Jose, CA, USA) and the entire mixture was inoculated subcutaneously (s.c.). Tumour growth was monitored once a week by measuring the tumour diameter with a caliper. Tumour volume (TV) was calculated as TV (mm^3^) = (*d*^2^ × *D*) / 2, where *d* is the shortest diameter and *D* is the longest diameter. Mice were sacrificed after 10 weeks of experiment by cervical dislocation following light anaesthesia by isoflurane inhalation. Samples were collected in 10% buffered formalin and were subjected to histological studies.

### Vector construction, virus production, transfections and transductions

The cDNA for P-selectin, amplified using the pCMV6-Entry vector containing P-selectin cDNA (2490 bp; OriGene, Rockville, MD, USA) as a template and primers (Additional file 1: Table S1), was cloned into the pSG5 plasmid (Agilent Technologies, Palo Alto, CA, USA). The resulting pSG5/SELP vector was used together with the pSV2neo vector (Invitrogen, Carlsbad, CA, USA) to co-transfect CHO-Pro5 cells using polyethylenimine (Sigma-Aldrich, Buchs, Switzerland). G418-resistant colonies were screened for the presence of P-selectin by flow cytometry.

For generation of the GAL3ST1-expressing vector, the human GAL3ST1 cDNA was amplified by PCR from the MCF7 cDNA library using the primers presented in Additional file 1: Table S1. The resulting insert was cloned into a pRRL-CMV-IRES-PURO vector as described previously [18] and named here as pRRL-CMV-GAL3ST1-IRES-PURO. For lentivirus production, packaging LentiX 293 T cells were co-transfected at 50–60% confluence with 20 μg of expression or control vector, 10 μg pMDL-g/p-RRE, 5 μg pRSV-REV and 5 μg pMk-VSVG (kindly provided by Dr D. Trono, École Polytechnique Fédérale de Lausanne, Switzerland) using polyethylenimine (Sigma-Aldrich). The production of virus particles and transduction of cells have been described previously [14].

For silencing *GAL3ST1*, a GAL3ST1 CRISPR/Cas9 knockout plasmid encoding the Cas9 nuclease and GAL3ST1-specific 20 nucleotide guide RNA (gRNA-AGTGATCCGGGCCAACGGCT) was purchased from Applied Biological Materials Inc. (Richmond, BC, Canada). For lentivirus production the same procedure as already described was used [14]. *GAL3ST1* knockout cells were selected with puromycin (1 μg/ml). Antibiotic-resistant cells were detached by trypsinisation and subcloned using a limiting dilution technique.

### Real-time PCR

Purification of RNA from tissues and cells was performed using the RNeasy Mini Kit (Qiagen, Hilden, Germany) according to the manufacturer’s instructions. The SuperScript RT (Thermo Fisher Scientific) was used to synthesise cDNA. The relative amounts of GAL3ST1 were determined by real-time PCR assay (qPCR) with iQ™ SYBR Green Supermix (Bio-Rad, Hercules, CA, USA) according to the manufacturer’s protocol, using iQ5 Optical System (Bio-Rad). β-actin was used as a reference gene. The primers used for the real-time PCR assay are presented in Additional file 1: Table S1. Gene expression was calculated using the ΔΔCt method [19].

### Western blotting analysis

Western blotting analysis was performed as described previously [18]. The antibodies used are presented in Additional file 2: Table S2.

### Flow cytometry

Flow cytometry with specific antibodies was performed as described previously [20]. The antibodies used are presented in Additional file 2: Table S2. For analysis of tumour cell–platelet aggregates, tumour cells and platelets were stained, respectively, with lipophilic fluorescence dyes DiD (red fluorescence) and DiO (green fluorescence) (Thermo Fisher Scientific) for 1 h at 37 °C. After washing, tumour cells and platelets were mixed and incubated for another 1 h at 37 °C. Cells stained with antibodies or tumour cell–platelet aggregates were resuspended in PBS and analysed using the BD FACS Canto II instrument (Becton*-*Dickinson). Data were processed and analysed using Flowing Software version *2.*

### Purification of gangliosides and neutral glycolipid, thin-layer chromatography and thin-layer chromatogram binding assay (TLC binding assay)

Gangliosides and neutral glycolipids were purified as described previously [21]. Glycolipids were extracted from 10^8^–10^9^ cells using the chloroform–methanol extraction method. HP-TLC was performed on Silica Gel 60 high-performance TLC (HPTLC) plates (Merck Millipore). For ganglioside/sulfatide separation, a chloroform:methanol:0.2% CaCl_2_ solvent system (60:40:9, v/v/v) was used, while neutral glycolipids were separated in a 2-isopropanol:methyl acetate:15 M ammonium hydroxide:water solvent system (75:10:5:15, v/v/v/v). The glycolipids were visualised by spraying the plate with primuline reagent (0.05% primuline in acetone/water, 4:1 by volume) and heated for 1 min at 120 °C.

Sulfatide and GalCer were detected by TLC binding assay primarily as described previously [14]. The antibodies used are presented in Additional file 2: Table S2.

### Hydrophilic interaction liquid chromatography coupled with electrospray mass spectrometry (HILIC-ESI-MS/MS)

Hydrophilic interaction liquid chromatography (HILIC) separations were carried out on the Dionex Ultimate 3000 RSCL chromatographic system (Thermo Scientific) equipped with an Atlantis HILIC column (Waters, Saint-Quentin, France). The system used 10 mM ammonium acetate with the addition of 0.1% formic acid (solution A) and acetonitrile with 0.1% formic acid (solution B) as mobile phases. The column was operated at 30 °C at a flow rate of 0.3 ml/min. The gradient started at 95% solution B for the first 0.5 min and went down to 50% solution B at 6.5 min and back up to 95% solution B at 7.5 min. The column was then stabilised for another 5 min. The chromatographic system was coupled to the maXis impact q-TOF mass spectrometer (Bruker Daltonics, Billerica, MA, USA) with the electrospray ion source operated in positive ion mode. The spectrometer parameters were set as follows: mass range 50–1300 m/z, spectra rate 4 Hz, nebulising gas pressure 1.5 bar, drying gas flow 8 L/min, capillary voltage 4500 eV, source energy 5 eV and collision energy 10 eV. To obtain the fragmentation spectra of the standards, the auto-MS/MS function was set for the three most abundant peaks. For the SIM experiment, the broadband collision-induced dissociation (bbCID) fragmentation was set at an energy of 35 eV. Three biological replicates of ceramide extracts were run for each examined cell line.

### Cell proliferation assay (SRB assay)

Cells (5 × 10^3^ cells) were grown in individual wells of 96-well plates (Greiner, Germany) in complete αMEM. After 24, 48, 72 and 96 h, the cells growing in successive wells were fixed in 10% trichloroacetic acid for 30 min at 4 °C, washed with water and dried. Fixed cells were incubated with 0.4% sulforhodamine B (SRB; Sigma-Aldrich) in 1% acetic acid for 20 min at RT. After washing with 1% acetic acid, the protein-bound dye was extracted with 10 mM Tris. Absorbance at 492 nm was measured on an EnSpire 2300 Multilabel Reader (Perkin-Elmer, Waltham, MA, USA). Data are presented as mean ± SD from two independent assays.

### Apoptotic assays

Caspase-3 activation was determined using the CaspGLOW fluorescein active caspase-3 staining kit (BioVision, Milpitas, CA, USA) according to the manufacturer’s instructions. The cells were seeded in six-well plates (Greiner) at a density of 5 × 10^5^ cells/ml. The following day, the cells were treated with three different concentrations (0.1, 0.5 and 1 μM) of doxorubicin hydrochloride (Pfizer, New York, NY, USA) for 48 h. The cells previously harvested by trypsinisation and centrifuged at 300 × *g* for 5 min were incubated with 1 μl of FITC-DEVD-FMK peptide for 45 min at 37 °C in an incubator with 5% CO_2_. The cells were then washed twice before being subjected to FACS analysis. Fluorescence was measured in FL-1 on the BD FACSCalibur (Becton*-*Dickinson). Data were processed and analysed using Flowing Software version 2.

Apoptosis was also measured by FITC-Annexin V and propidium iodide (PI) staining using the FITC Annexin V Apoptosis Detection Kit (Becton*-*Dickinson) according to the manufacturer’s instructions. Cells were subjected to fluorescence analysis using the BD FACSCalibur *(*Becton*-*Dickinson) and Flowing Software version 2. The percentage of Annexin V-positive cells corresponded to cells in early apoptosis, while Annexin V and PI-positive cells corresponded to cells in late apoptosis.

### Flow adhesion assay

Adhesion under laminar flow conditions of human breast cancer MDA-MB-231 cells to P-selectin-expressing or E-selectin-expressing CHO cells was quantified using a parallel plate flow chamber [22]. P-selectin-expressing CHO-Pro5 cells, E-selectin-expressing CHO cells and control CHO cells were grown to confluence in the presence of complete αMEM on Permanox™ eight-well chamber slides (Nunc, Roskilde, Denmark). On the day of the experiment, the slides containing a monolayer of cells were assembled in the flow chamber (250 μm gap thickness; Immunetics, Boston, MA, USA), and placed on the stage of an inverted microscope (NiconN 2000TS, Tokyo, Japan) equipped with 200× objective lenses.

In the first stage, P-selectin-expressing or E-selectin-expressing CHO cells and control CHO cells were subjected to a fluid shear flow of 1 dyn/cm^2^ formed by an infusion PHD 2000 pump (Harvard Apparatus, Holliston, MA, USA) for 5 min, with complete αMEM medium at room temperature. In the second stage (adhesion assay), human breast cancer cells were suspended in αMEM medium and stained with CellTracker dye (Molecular Probes, Eugene, OR, USA) at a concentration of 0.5 mg/ml for 1 h at 37 °C. After washing, cancer cells (10^6^/ml cells) were re-suspended in complete αMEM, injected into the flow chamber and allowed to roll on CHO cell monolayers. Interacting cells were defined as those that rolled in the field of view. Those cells were quantified in a single 200× field of view of 0.2 mm^2^, during a 5-s perfusion. Fluid shear flow was changed every 5 min in increasing order of 0.15, 0.3, 0.6, 1.0, 1.5, and 3 dyn/cm^2^. Data were processed using Iris software (MEDI.COM, Wroclaw, Poland).

### Platelet isolation and activation

Human platelets were isolated from whole blood according to the Springer Lab Protocol (Springer Lab separation of platelets from whole blood by Azucena Salas, Springer Lab, The CBR Institute for Biomedical Research, Inc., Boston, MA, USA). Briefly, peripheral blood from healthy volunteer donors was collected into citrate solution (BD Biosciences). Tubes were centrifuged and the upper phase, platelet-rich plasma (PRP), was transferred to a new tube containing ACD buffer (6.25 g sodium citrate⋅2 H_2_O, 3.1 g citric acid anhydrous, and 3.4 g d-glucose in 250 ml H_2_O) at a ratio of 9:1. The sample was centrifuged again at 900 × *g* for 5 min at RT and the platelet pellet was resuspended in 1 ml of Hepes-Tyrode buffer. Freshly isolated platelets were used within 2 h.

Platelets (10^6^ platelets/ml), resuspended in Hepes-Tyrode buffer, were activated by incubation for 5 min at 37 °C with 1 μM or 5 μM ADP (Sigma-Aldrich) in ultrapure DEPC water. P-selectin expression on the platelet surfaces, analysed by flow cytometry, was used as an indicator of activation. Platelets that were not activated served as negative controls. Only single platelet suspensions, distinguished from platelet aggregates by their light-scattering properties, were subjected to flow cytometry analysis.

### Platelet aggregation assay

Platelet aggregation was monitored on a U-5100 UV-Vis Ratio-Beam Spectrophotometer (Hitachi, Ibraki Prefecture, Japan) via the modified turbidimetric method described originally by Born [23]. Platelets (10^6^ platelets/ml) suspended in Hepes-Tyrode buffer were activated with 1 μM or 5 μM of ADP and the formation of aggregates was analysed at 37 °C for 1 min with constant stirring (100 × *g*) for 500 s. The spectrophotometer was calibrated with an unstimulated suspension of platelets (10^6^ platelets/ml) in Hepes-Tyrode buffer representing 0% aggregation, with the suspension of activated platelets (10^6^/ml) in Hepes-Tyrode buffer (platelet-rich plasma, PRP) representing 100% aggregation [24].

The effect of sulfatide on the aggregation of platelets was studied by incubating sulfatide-expressing breast cancer cells (10^6^ cells/ml) and platelets (10^6^ platelets/ml) in a ratio of 1:1, at 37 °C for 1 min with constant stirring (100 × *g*) in the presence of ADP (1 μM and 5 μM) for 500 s.

### Evaluation of immunohistochemistry reactions

The immunohistochemistry (IHC) reactions were evaluated by two independent pathologists using a BX-41 light microscope (Olympus, Tokyo, Japan). Expression of GAL3ST1 in tumour cells was evaluated using the semi-quantitative immunoreactive score (IRS) according to Remmele and Stegner [25]. The scoring system comprised the percentage of positively stained cells (0, no reaction; 1, < 10%; 2, 11–50%; 3, 51–80%; and 4, > 81% cells), as well as the intensity of staining (graded as 0 = no reaction, 1 = weak, 2 = moderate and 3 = strong staining). The lack of IHC GAL3ST1 expression (0 points using the IRS scale) was considered negative, and 1–12 points using the IRS scale was considered positive expression. The median of IRS GAL3ST1 expression was established as the cut-off point for the aforementioned analysis. The same cut-off points (0 vs 1–12) was considered in the analysis of survival.

### Terminal transferase dUTP nick end labelling assay

The apoptotic terminal transferase dUTP nick end labelling (TUNEL) assay was performed using the ApopTag® Peroxidase In Situ Apoptosis Detection Kit (Merck Millipore). Paraffin sections were de-waxed in xylene, rehydrated in alcohol, rinsed in distillate water and washed with PBS (pH 7.4). The sections were then incubated with Proteinase K (Dako) for 5 min at RT and rinsed in PBS. Endogenous peroxidase was blocked by incubation in 3% H_2_O_2_/PBS for 5 min. Next, the sections were incubated, first with Equilibration Buffer for 10 min at RT and then with TdT enzyme and reaction buffer at 37 °C for 1 h. The reaction was stopped using a stop buffer, and anti-digoxigenin peroxidase-conjugated antibodies were applied for 30 min at RT. To visualise the TUNEL-positive cell nuclei, the sections were incubated for 10 min with diaminobenzidine (Dako). Finally, the sections were counterstained with Mayer’s haematoxylin and after dehydration in alcohols mounted in SUB-X Mounting Medium (both Dako). TUNEL-positive cell nuclei expression in tumour cells was evaluated using a BX-41 light microscope equipped with the computer-assisted image analysis program Cell^D^ (Olympus). Three fields with the highest number of tumour cells yielding positive reaction (hot spots) were selected for every stained section and then analysed. The general result for every section was the average of the three hot-spot percentages of cells showing a brown reaction product.

### Statistical analysis

All statistical analyses were performed using Prism 5.0 (GraphPad, La Jolla, CA, USA) and Statistica 10 (StatSoft Inc. Tulsa, OK, USA). The Shapiro–Wilk test was used to evaluate the normality assumption of examined groups. The paired *t* test was used for statistical analysis of in-vivo tumour growth experiments. The Mann–Whitney test was used to compare the groups of data that did not meet the assumptions of the parametric test. Additionally, the Spearman correlation test was used to analyse the existing correlations. The Kaplan–Meyer method was used to construct survival curves. To evaluate the survival analysis, the Mantel–Cox test was performed. A Cox proportional hazards model with forward stepwise selection was used to calculate univariate and multivariate hazard ratio for the studied parameters. In all analyses, the results were considered statistically significant when *p* < 0.05.

To statistically evaluate the significance of differences between Cer and HexCer levels obtained by MS analysis, a one-way analysis of variance (ANOVA) test was performed. The *p*-value significance threshold was set at 0.01 due to a relatively low number of measured replicates.

## Results

### Accumulation of sulfatides in breast cancer cells correlates with increased sensitivity to doxorubicin-induced and hypoxia-induced apoptosis

**Fig. 1:**
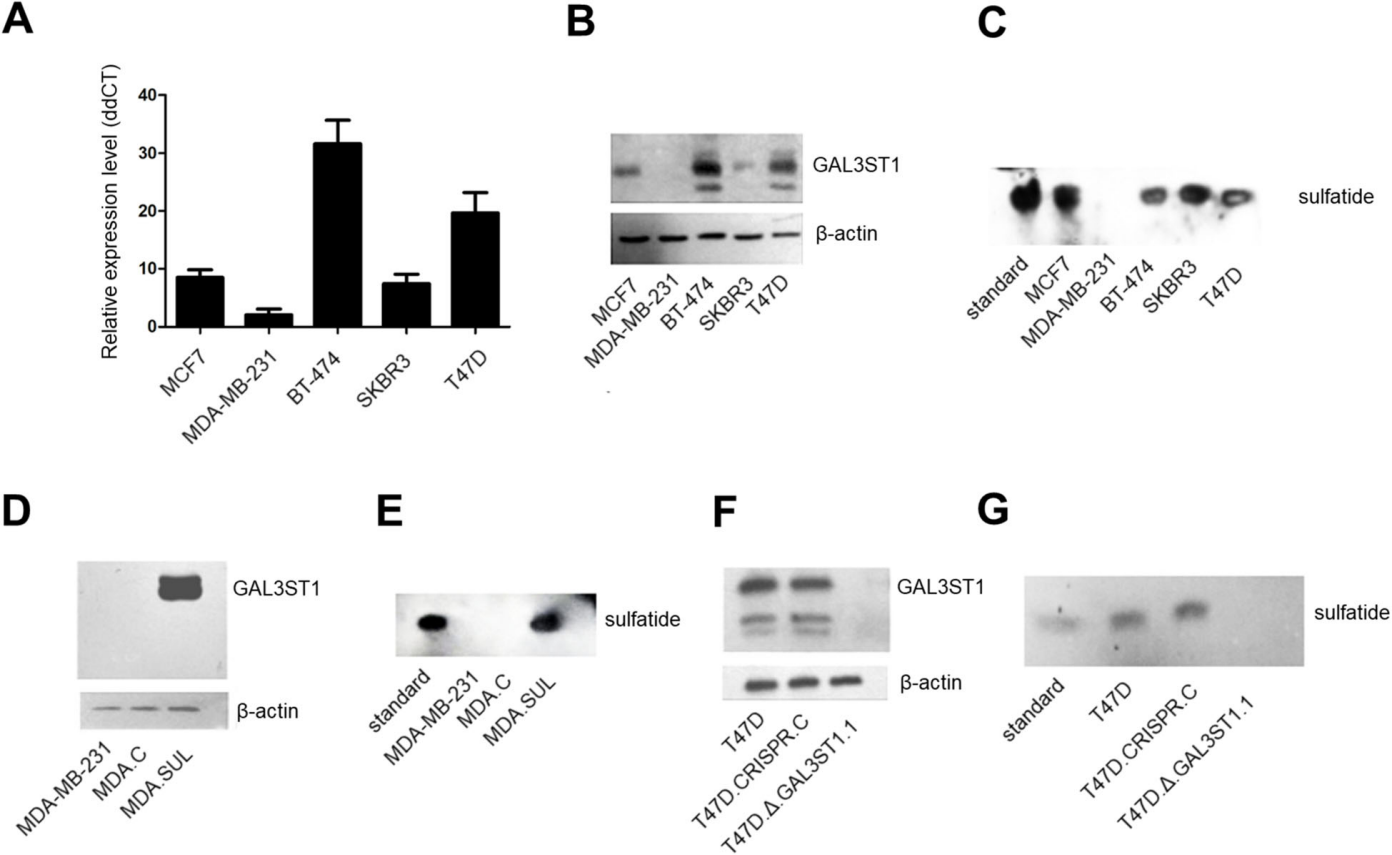
Expression of GAL3ST1 and sulfatide in breast cancer cell lines and characteristics of human breast cancer cell lines with overexpression or blocked expression of GAL3ST1 and sulfatide. **a** Expression of GAL3ST1 mRNA in breast cancer cell lines. Real-time PCR used to analyse GAL3ST1 mRNA. *GAL3ST1* expression levels normalised against β-actin and MDA-MB-231 cells served as calibrator sample. Results expressed as mean. **b** Western blotting analysis of GAL3ST1 expression in breast cancer cell lines. Anti-GAL3ST1 rabbit polyclonal antibodies used to detect GAL3ST1 in cell lysates. **c** Immunostaining of gangliosides from human breast cancer cell lines, separated by HP-TLC, with mouse monoclonal antibody against sulfatide. **d** Western blotting analysis of GAL3ST1 expression in parental MDA-MB-231 cells, control MDA-MB-231 cells (MDA.CTR) and MDA-MB-231 cells overexpressing sulfatide (MDA.SUL). **e** Immunostaining of gangliosides from MDA-MB-231, MDA.CTR and MDA.SUL cells. **f** Western blotting analysis of GAL3ST1 expression in parental T47D cells, control T47D cells (T47D.CRISPR.C) and T47D cells with knockout of *GAL3ST1* (T47D.Δ.GAL3ST1.1). **g** Immunostaining of gangliosides from T47D, T47D.CRISPR.C and T47D.Δ.GAL3ST1.1 cells. For western blotting, 40 μg of proteins separated by SDS-PAGE under reducing conditions on a 12% gel and electrophoretically transferred onto a nitrocellulose membrane. β-actin served as internal control. For immunostaining, aliquots of total gangliosides corresponding to 1 × 10^7^ cells applied to HP-TLC plate. Gal3ST1 galactosylceramide sulfotransferase

We have previously shown that galactosylceramide (GalCer) increases the resistance of breast cancer MDA-MB-231 cells to doxorubicin-induced apoptosis [14]. Since GalCer is a precursor molecule in the synthesis of sulfatide, we wondered how the conversion of GalCer to sulfatide would affect the apoptotic properties of breast cancer cells. First, we examined the expression of GAL3ST1 and sulfatides in breast cancer MCF7, T47D, SKBR3, BT-474 and MDA-MB-231 cell lines. Neither galactosylceramide sulfotransferase nor sulfatides were detected in the metastatic MDA-MB-231 cells (Fig. 1a–c). Based on these results, MDA-MB-231 cells were used to create a model with overexpression of sulfatide, representing a gain-of-function phenotype. On the other hand, T47D cells with high expression of GAL3ST1 were chosen to obtain cells with inhibited synthesis of sulfatide, representing a loss-of-function phenotype. Transduction of MDA-MB-231 cells with the pRRL-CMV-GAL3ST1-IRES-PURO expression vector containing cDNA for GAL3ST1 resulted in a cell population that overexpressed sulfotransferase and sulfatide (Fig. 1d, e and Additional file 3: Figure S1A). These cells that had the same morphology and growth rate as appropriate control cells were called MDA.SUL cells (Additional file 3: Figure S1B).

In order to stably and irreversibly inhibit the expression of GAL3ST1 and therefore block the synthesis of sulfatides, we created a CRISPR-edited knockout of *GAL3ST1* in T47D cells. After transfection and selection with puromycin, 20 resistant clones were isolated, propagated and screened for the absence of GAL3ST1. Western blotting analysis demonstrated that three of these clones lacked GAL3ST1 expression on the protein level. Since they had the same morphology and proliferation rate, one of the clones was randomly chosen, named T47D.Δ.GAL3ST1.1, and used in further studies (Fig. 1f and Additional file 3: Figure S1C, D). Immunostaining of gangliosides purified from T47D.Δ.GAL3ST1.1 cells with anti-sulfatide antibody revealed the absence of sulfatide in these cells (Fig. 1g). A clone of T47D cells transduced with the control CRISPR/Cas9 plasmid encoding the Cas9 nuclease and non-specific 20 nt guide RNA (gRNA), called T47D.GAL3ST1.C, was obtained using the same approach.

**Fig. 2:**
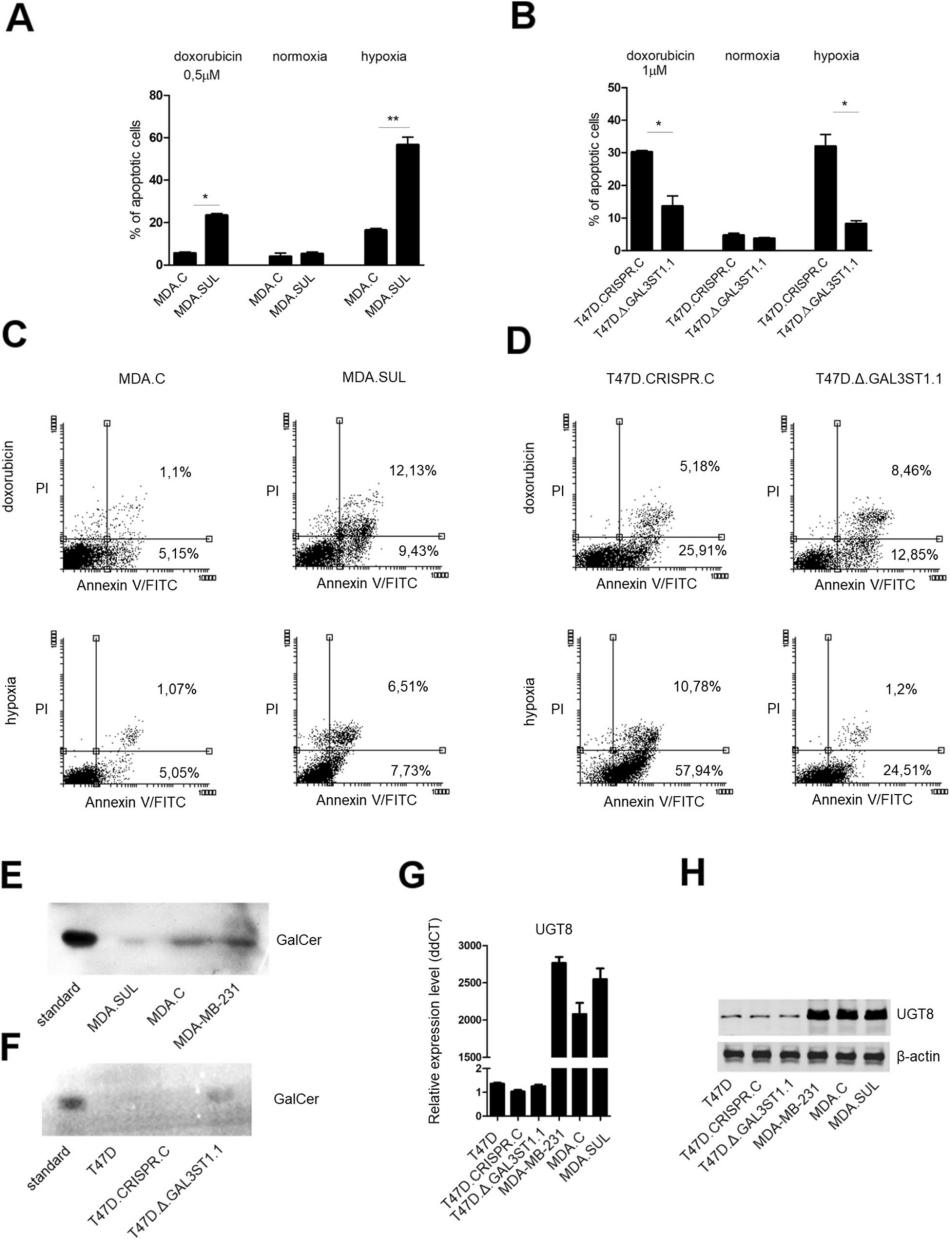
Sensitivity of breast cancer MDA-MB-231 and T47D cells with varying expression of GAL3ST1 to apoptosis induced by doxorubicin and hypoxia. Cells grown in presence of doxorubicin at concentration of 0.5 μM (MDA-MB-231 cells) or 1 μM (T47D) and in hypoxic conditions (1% O_2_) for 48 h. Cellular response measured by presence of active form of caspase-3 (**a, b**) or staining with Annexin V and propidium iodide (**c, d**). Statistically significant differences (**p < 0.01, *p <0.05). Flow cytometry dot plots show percentage of early apoptotic cells (Annexin V^+^/PI^−^, lower right) and late apoptotic cells (Annexin V^+^/PI^+^, upper right). Levels of galactosyloceramide (GalCer) and UGT8 in MDA-MB-231 and T47D cells with varying expression of GAL3ST1. Immunostaining of neutral glycolipids from MDA-MB-231, MDA.C and MDA.SUL cells (**e**) and from T47D, T47D*.*CRISPR*.*C and T47D.Δ.GAL3ST1.1 cells (**f**) separated by HP-TLC. For immunostaining, aliquots of neutral glycosphingolipids corresponding to 1 × 10^7^ cells applied to HP-TLC plate and stained with anti-GalCer rabbit polyclonal antibodies. **g** Real-time PCR used to analyse expression of UGT8 mRNA in MDA-MB-231 and T47D cell lines with varying expression levels of sulfatide. UGT8 levels normalised against β-actin and T47D.CRISPR.C cells assigned as calibrator sample. Results expressed as mean. **h** Western blotting analysis of UGT8 expression in breast cancer cell lines with different expression levels of sulfatide. Anti-UGT8 rabbit polyclonal antibodies used to detect UGT8 in cell lysates. FITC fluorescein isothiocyanate, GalCer galactosyloceramide, PI propidium iodide

To assess whether the accumulation of sulfatides can affect the sensitivity of breast cancer cells to drug-induced apoptosis, MDA.SUL and control MDA.C cells were incubated with doxorubicin for 48 h. We found that after doxorubicin treatment, the percentage of apoptotic cells was 4.2-fold higher in MDA.SUL cells than in MDA.C cells (*p* < 0.05) (Fig. 2a). We also analysed the sensitivity of modified cells to apoptosis under hypoxic conditions and observed that the percentage of apoptotic MDA.SUL cells was 3.5-fold higher than for MDA.C cells (*p* < 0.01) (Fig. 2a**)**. We confirmed these results through the analysis of the loss-of-function phenotype. When T47D.Δ.GAL3ST1.1 cells and control T47D cells were treated with doxorubicin or subjected to hypoxic conditions, control T47D.GAL3ST1.C cells were more sensitive to apoptosis than T47D.Δ.GAL3ST1.1 cells by 2.2-fold (*p* < 0.05) and 3.8-fold (*p* < 0.05), respectively (Fig. 2b). The results obtained using the CaspGLOW fluorescein active caspase-3 staining kit were further confirmed through staining the apoptotic cells with FITC-Annexin V and PI. In the gain-of-function cellular model, the percentage of all (early and late) apoptotic cells was higher in MDA.SUL cells than in MDA.C cells by 3.4-fold after doxorubicin treatment and by 2.3-fold in hypoxic conditions (Fig. 2c). However, in doxorubicin-treated and hypoxia-treated T47D.Δ.GAL3ST1.1 cells, representing the loss-of-function cellular model, the percentage of all (early and late) apoptotic cells decreased compared to control T47D cells by 1.5-fold and 2.7-fold, respectively (Fig. 2d). Based on these results we questioned whether the pro-apoptotic effects of sulfatide accumulation could be linked to decreased amounts of anti-apoptotic GalCer [14] since the latter is the substrate for sulfotransferase. Immunostaining of neutral glycosphingolipids purified from MDA.SUL cells revealed highly decreased binding of anti-GalCer antibodies to HP-TLC plates compared to neutral glycosphingolipids from MDA.C and parental MDA-MB-231 cells (Fig. 2e). These results obtained using MDA.SUL cells were further supported by our second cellular model represented by T47D cells with knockout of the *GAL3ST1*. In such cells, an increased level of GalCer compared to control T47D.GAL3ST1.C and wild-type T47D was observed as a consequence of blocked sulfatide synthesis (Fig. 2f). These data raised the possibility that the reduced level of GalCer is a result of decreased expression of UGT8 or of increased conversion of GalCer to sulfatide caused by overexpression of GAL3ST1. Therefore, we analysed the expression of the *UGT8*. We did not find any differences between analysed cells at the mRNA or protein level (Fig. 2g, h), which strongly suggests that changes in GalCer levels are associated with enhanced synthesis of sulfatide.

### Sulfatide inhibits tumour growth

**Fig. 3:**
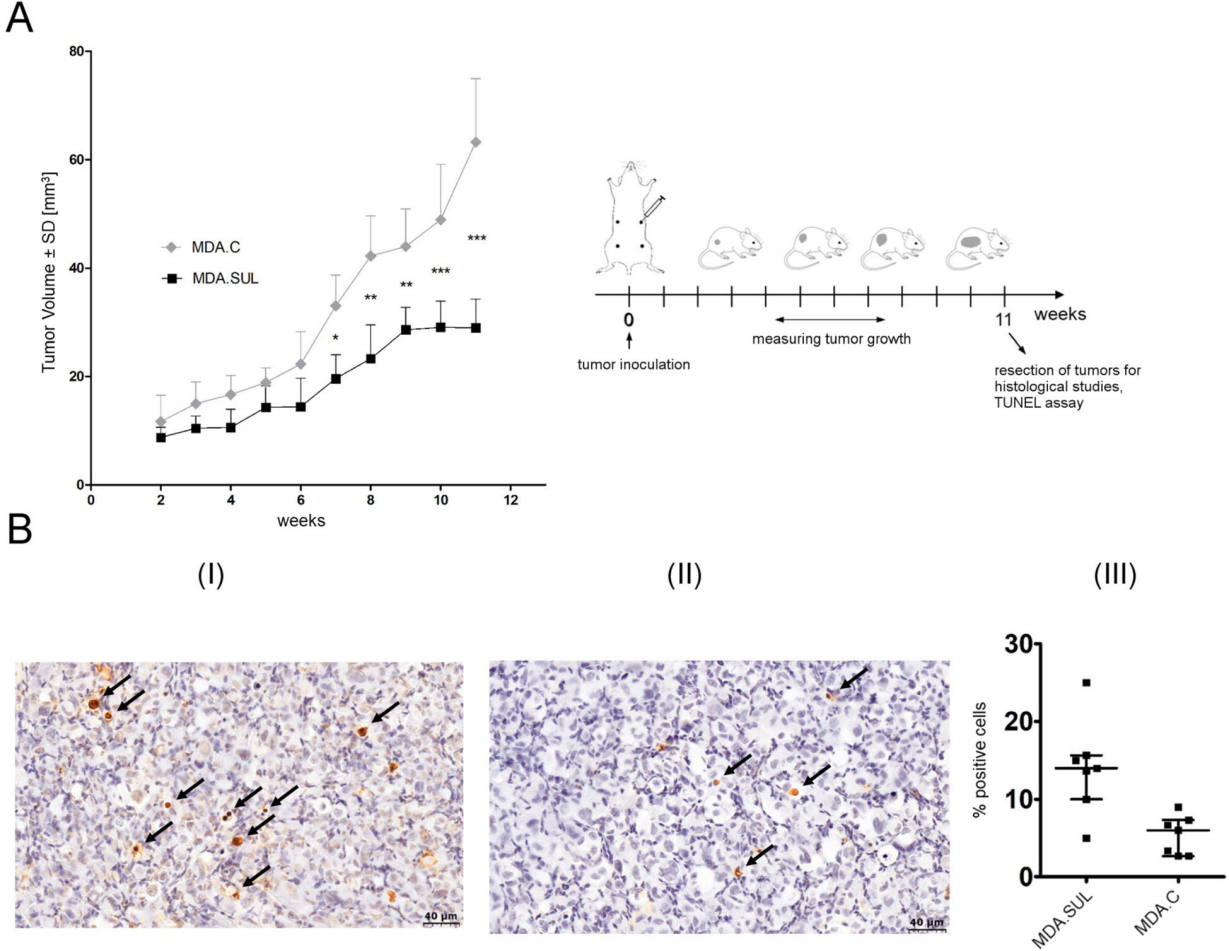
Xenograft tumour growth of MDA.SUL cells with overexpression of GAL3ST1 and control MDA.C cells. **a** Tumour growth recorded once a week by measuring diameter with a calliper. Data shown as mean tumour volume for group of mice (*n*   =  8 for MDA.SUL cells and *n* = 9 for MDA.C cells) ± SE at each indicated time point. Data analysed using Bonferroni multiple comparison test. ^*^p < 0.05, ^**^p < 0.01, ^***^p < 0.001. **b** TUNEL assay after subcutaneous implantation of MDA.SUL (I) and MDA.C (II) cells. Arrows indicate the apoptotic cells. Numbers of apoptotic cells in MDA.SUL and MDA.C tumours compared (III) by Mann–Whitney *U* test (*p* = 0.0494). TUNEL terminal transferase dUTP nick end labelling

To determine whether the increased synthesis of sulfatides can affect the malignant phenotype of breast cancer cells, MDA.SUL and MDA.C cells were subcutaneously injected into nude mice. Statistically significant reductions in tumour volumes were observed in MDA.SUL tumours compared to tumours formed by MDA.C cells (Fig. 3a). In week 11, the mean volume of tumours developed by MDA.SUL cells was 28.95 mm^3^, and the mean volume of tumours formed by MDA.C cells was 63.26 mm^3^. Since the expression of sulfatide in breast cancer cells affects their sensitivity to apoptosis (see earlier), the apoptotic properties of MDA.SUL and MDA.C cells were analysed in vivo by TUNEL assay (Fig. 3b). Increased numbers of apoptotic cells were found in MDA.SUL tumours (Fig. 3b, I) compared to MDA.C tumours (Fig. 3b, II) (*p* = 0.0494, Mann–Whitney *U* test).

### The anti-apoptotic effects of GalCer are not associated with changes in ceramide levels

**Fig. 4:**
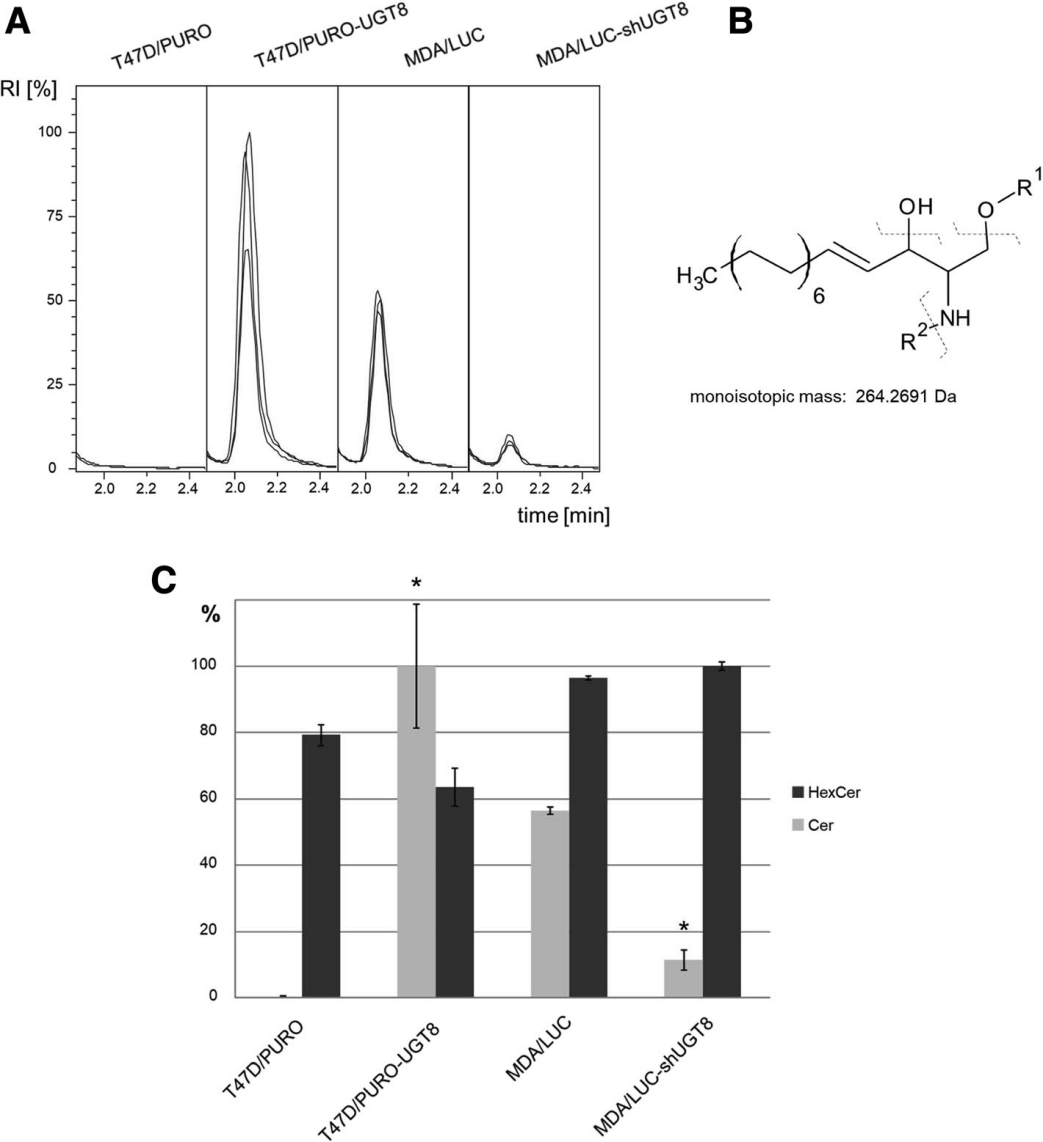
Relative quantification of ceramides in selected cell lines. **a** Extracted ion chromatograms for 264.269 ± 001 m/z in cell line samples with peak at RT = 2.1 min corresponding to HexCer. RI stands for Relative Intensity. **b** Structure of reporter ion selected for relative quantification. **c** Relative quantities of Cer (dark grey) and HexCer (light grey) in breast cancer cell lines. 100% value corresponds to highest concentration of particular analyte. Error bars represent standard deviations of three biological replicates. Statistically significant differences (*p < 0.01) in analyte levels

We hypothesised previously that the anti-apoptotic effects of GalCer in breast cancer cells with high expression of UGT8 are associated with decreased levels of pro-apoptotic ceramide acting as a cellular regulator controlling cell fate by promoting cell survival [14, 15]. To quantify the amounts of ceramide in breast cancer cells with high and low expression of UGT8, we modified and applied a previously described HILIC-MS/MS methodology [26] involving monitoring of the characteristic ion at 264 m/z, a product of fragmentation of both Cer and HexCer (hexosyl-ceramide) but not oligosaccharide substituted ceramides. This method allowed us to relatively quantify Cer and HexCer in the following cell lines: control MDA/LUC cells with high expression of UGT8 and MDA/LUC-shUGT8 cells with suppressed expression of UGT8 [14], control T47D/PURO cells with no expression of UGT8 and T47D/PURO-UGT8 cells with overexpression of UGT8 (Additional file 3: Figure S1E, F). We found that HexCer levels are affected by the presence or absence of UGT8 as expected, while Cer levels do not differ significantly (Fig. 4). It must be noted that the concentration of free ceramide is almost two orders of magnitude greater than the concentration of HexCer. This may explain why a major alteration of HexCer levels does not significantly influence the Cer pool. Moreover, an almost complete absence of signals corresponding to hexosyl-ceramide in the T47D/PURO cell line, naturally not expressing UGT8, and highly decreased levels of hexosyl-ceramide-derived signals in MDA/LUC-shUGT8 cells with inhibited synthesis of GalCer strongly suggest that the HexCer pool consists mainly of galactosylceramide.

### Sulfatide is responsible for binding breast cancer cells to P-selectin-expressing CHO cells and activated human platelets

**Fig. 5:**
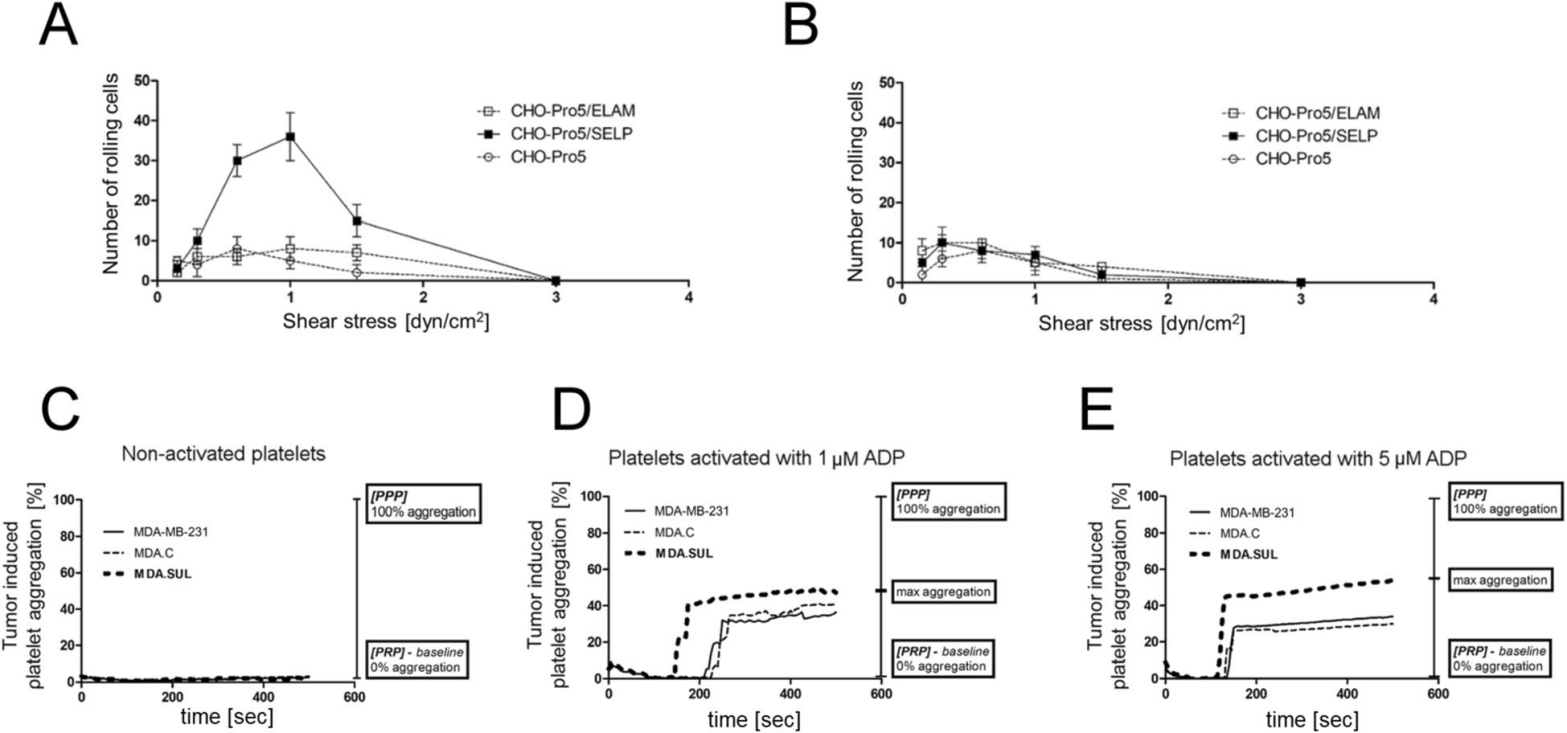
Binding of sulfatide-expressing (**a**) MDA.SUL cells and (**b**) MDA.C cells to P-selectin-expressing CHO-Pro-5 (CHO-Pro5/SELP) cells or E-selectin-expressing CHO-Pro-5 (CHO-Pro5/ELAM) cells and control CHO-Pro-5 cells under defined laminar flow conditions. Points on graphs represent mean numbers of breast cancer cells interacting with CHO-Pro-5 cells obtained from three independent experiments. Formation of heterotypic tumour cell–platelet aggregates by breast cancer MDA-MB-231 cells and activated platelets. Turbidimetric platelet aggregation assay with activated platelets and MDA-MB-231, MDA.C and MDA.SUL cells. Platelets (10^6^) and tumour cells (10^6^) in 1 ml of Tyrode’s buffer incubated in absence (**c**) or presence of 1 μM (**d**) or 5 μM ADP (**e**). ADP adensoine diphosphate, PPP platelet-poor plasma, PRP platelet-rich plasma

It was proposed that sulfatides present on the surface of breast cancer cells are P-selectin ligands [12]. However, there is a lack of direct experimental evidence supporting this hypothesis. Therefore, the binding of breast cancer cells with neoexpression of sulfatide to P-selectin-expressing cells was studied under physiologic flow conditions. Since selectin-expressing CHO cells are a good experimental model to study ligands for selectins [17, 27], their interaction with sulfatide-expressing MDA.SUL cells and control MDA.C cells was analysed. At applied fluid shear stresses of 0.6 and 1.0 dyn/cm^2^, which are similar to conditions found in microcirculation and postcapillary venules [28], MDA.SUL cells rolled in considerable numbers (30 and 36, respectively) on a monolayer of P-selectin-expressing CHO cells (Fig. 5a, Additional file 4: Figure S2A, Additional file 5: Video S1). In contrast, MDA.C cells did not interact with P-selectin-expressing CHO cells at any of the flow rates (Fig. 5b). Additional evidence for a specific interaction of breast cancer cells with P-selectin came from control experiments in which parental or E-selectin-expressing CHO cells were used (Additional file 4: Figure S2B, Additional file 6: Video S2). None of the analysed breast cancer cell lines interacted with such cells.

**Fig. 6:**
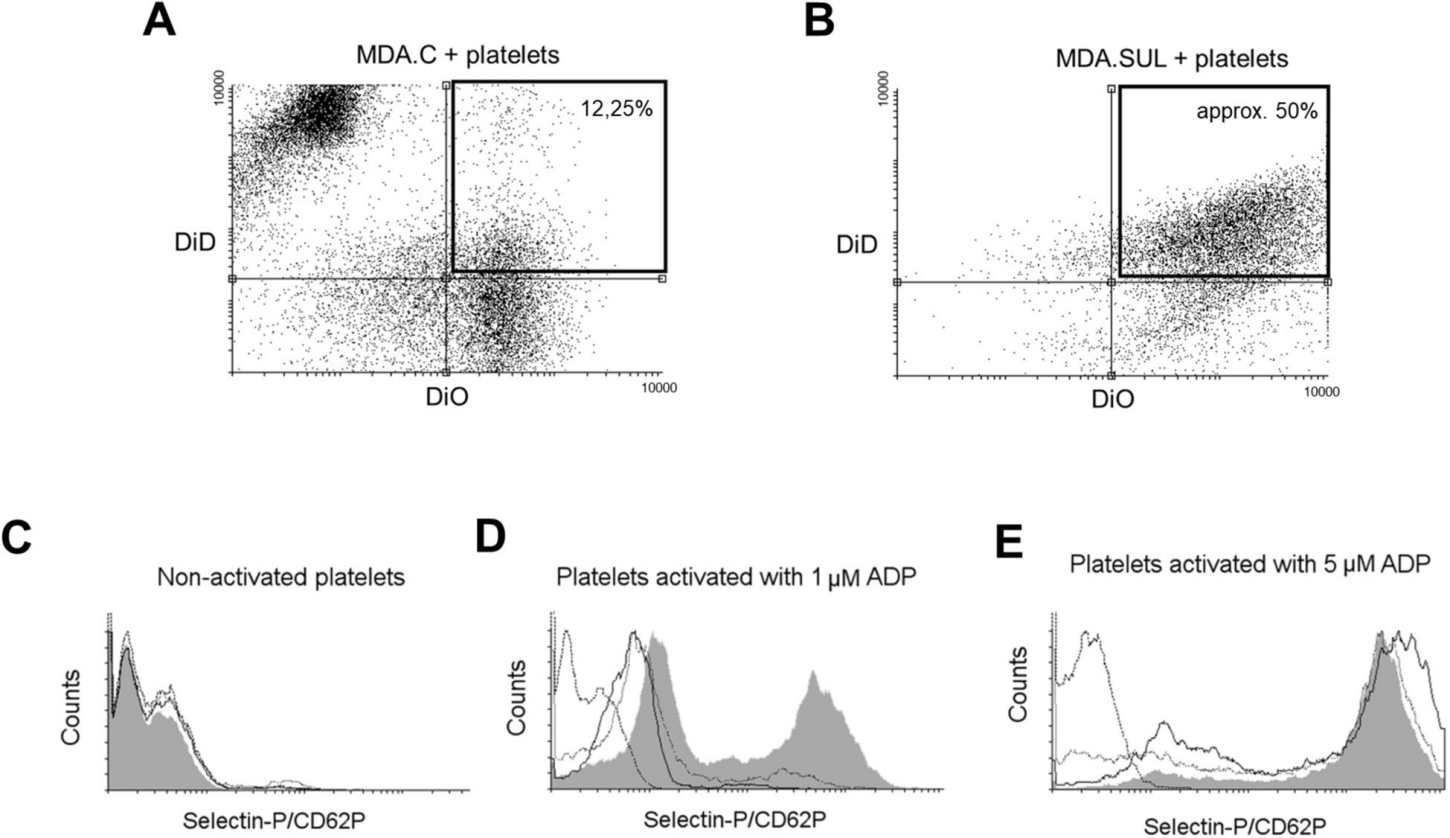
Flow cytometry analysis of tumour cell–platelet aggregates. **a** MDA.C and (**b**) MDA.SUL cells labelled with DiD (red) and platelets labelled with DiO (green) incubated for 1 h at room temperature. Box marks mixed aggregates formed by cancer cells and platelets. Sulfatides present on breast cancer cells stimulate activation of platelets. Flow cytometric analysis of P-selectin expression using monoclonal antibody against P-selectin human platelets (10^6^) (**c**) non-activated and activated with ADP at concentration of (**d**) 1 μM or (**e**) 5 μM incubated with (10^6^) parental breast cancer MDA-MB-231 cells (solid line), control MDA.C cells (dotted line) and MDA.SUL with overexpression of sulfatide (grey). Platelets incubated only with secondary antibodies used as control (dashed line). ADP adensoine diphosphate

The interaction between sulfatides and platelet P-selectin is critical for the formation of stable cancer cell–platelet aggregates, which in turn facilitates metastases [11, 29]. Here, we showed that breast cancer cells expressing sulfatide can aggregate activated human platelets in vitro, in a process called tumour cell-induced platelet aggregation (TCIPA). For this purpose, aggregation of ADP-activated platelets in the presence of MDA.SUL, MDA.C and parental MDA-MB-231 cells was examined. A significant effect of MDA.SUL cells on the aggregation of platelets after activation with 1 μM and 5 μM of ADP was observed compared to aggregation curves obtained for activated platelets incubated with MDA.C cells (Figs. 5c–e). These data suggest that increased aggregation of activated platelets is caused by the formation of heterotypic aggregates between platelets and tumour cells expressing sulfatide on their surface. The presence of such mixed aggregates was confirmed by flow cytometry. When breast cancer cells and platelets were labelled with fluorescence dyes DiD and DiO, respectively, considerable amounts of heterotypic tumour cell–platelet aggregates were found only in MDA.SUL cells (Figs. 6a, b).

### Sulfatides present on breast cancer cells increase the expression of P-selectin in activated platelets

Merten et al. [10] showed that sulfatide is not only a ligand for P-selectin, but its presence on the surface of platelets affects the activation of neighbouring platelets by increasing P-selectin expression. Therefore, we assessed the presence of P-selectin on the surface of activated platelets treated with ADP and found that treated platelets expressed high amounts of P-selectin (Additional file 7: Figure S3). Importantly, when activated platelets treated with 1 μM ADP were incubated with MDA.SUL cells, a subpopulation of cells with highly increased P-selectin expression was observed (Fig. 6d). Such a cell subpopulation was not observed when activated platelets were incubated with parental MDA-MB-231 and MDA.C cells. It should be stressed that the activated effect of sulfatide present on the surface of breast cancer cells was observed only in partially activated platelets since incubation of MDA.SUL cells with resting platelets has no effect on the surface expression of P-selectin. Incubation of ADP (5 μM) activated platelets demonstrating very high P-selectin expression levels with MDA.SUL cells did not increase P-selectin expression (Fig. 6e). Our data indicate that sulfatide expressed by cancer cells is not only a ligand for P-selectin, but also plays a role as a regulatory factor affecting P-selectin expression in activated platelets.

### Expression of GAL3ST1 in invasive ductal carcinoma and its correlation with patients’ clinical and pathological data

**Fig. 7:**
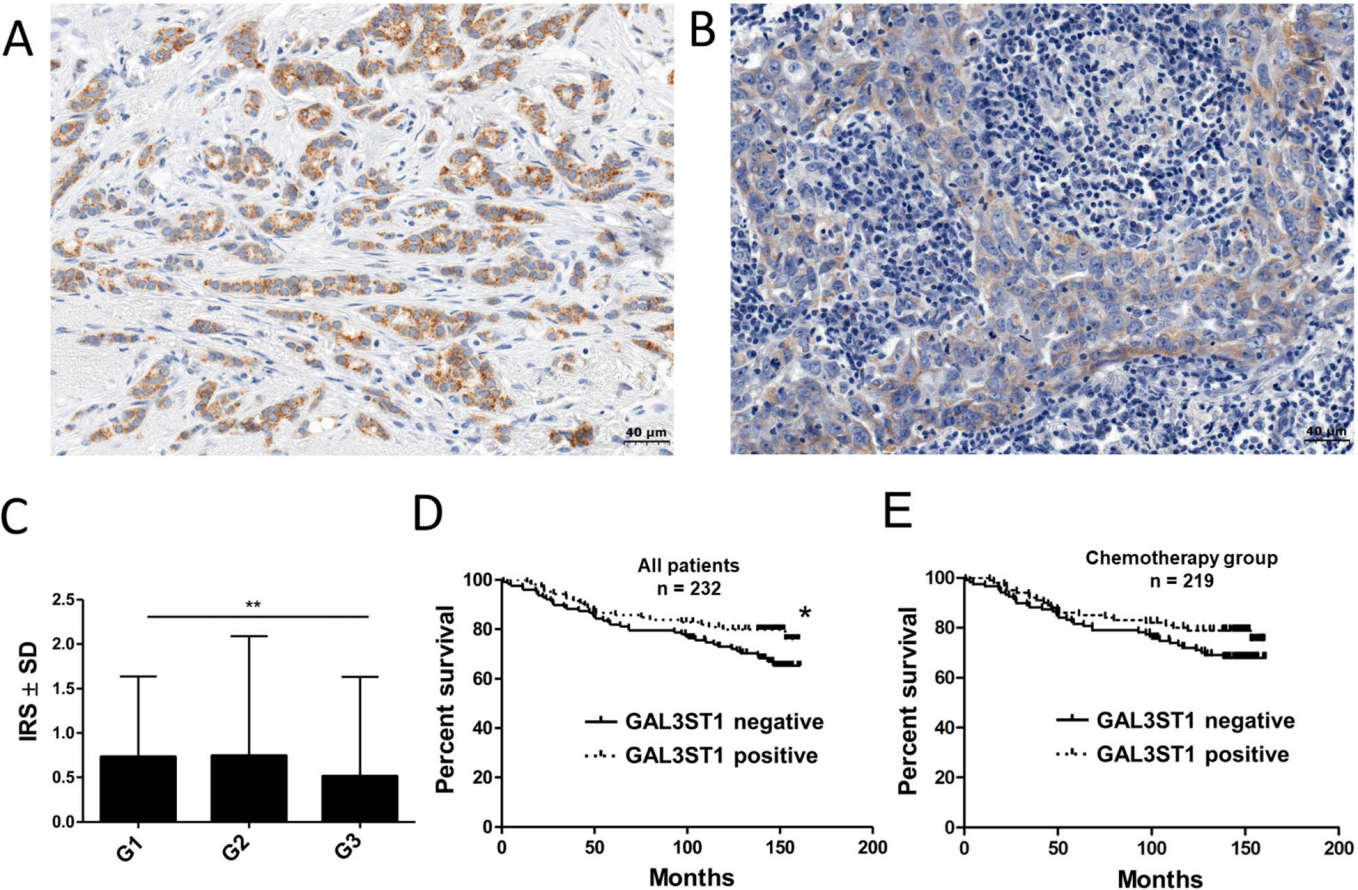
Expression of GAL3ST1 in IDC tissue specimens. Immunohistochemical staining of breast cancer tumours representing malignancy grades: (**a**) G1 and (**b**) G3 (magnification 400×). **c** Reaction intensities with anti-GAL3ST1 antibody calculated on basis of semi-quantitative IRS scale of Remmele and Stegner [21] and represented as mean. **p < 0.01 for G1 grade breast tumours compared to G3 grade breast tumours (Mann–Whitney *U* test). **d** Mantel–Cox analysis distinguished breast cancer patients who were GAL3ST1-positive and GAL3ST1-negative. Patients expressing GAL3ST1 had longer overall survival (*p < 0.05). **e** Overall survival of GAL3ST1-positive and GAL3ST1-negative breast cancer patients who received chemotherapy (Mantel–Cox analysis, *p* = 0.1206). Gal3ST1 galactosylceramide sulfotransferase, IRS semi-quantitative immune reactive score, SD standard deviation

The expression of GAL3ST1 in paraffin sections of invasive ductal carcinoma (IDC) specimens was studied using IHC (Fig. 7a, b). Positive cytoplasmic expression of GAL3ST1 was observed in tumour cells in 127 (54.74%) of the 232 examined cases. Higher expression of GAL3ST1 was observed in less aggressive G1 tumours than in highly aggressive G3 tumours (Mann Whitney test, *p* < 0.01; Fig. 7c). However, there were no significant associations with the expression of GAL3ST1 and other clinicopathological data (pTNM, stage, ER, PR and HER2 statuses, molecular subtypes or age at diagnosis).

To validate the predictive ability of GAL3ST1 positivity in primary breast tumours, the expression of GAL3ST1 was analysed in our cohort of breast cancer patients by IHC. Analysis of the survival rate revealed that GAL3ST1 expression in IDC cells is associated with longer overall survival (OS) (Fig. 7d, Table 2). Using cellular models, we have shown that high expression of GAL3ST1 is associated with increased sensitivity to doxorubicin-induced apoptosis. Accordingly, a similar type of analysis was performed only for breast cancer patients who received chemotherapy. We observed a correlation between GAL3ST1 immunoreactivity in cancer cells in these chemotherapy-treated breast cancer patients. However, this was not statistically significant with the overall survival rate (Fig. 7e, Table 2).

**Table 2:** Univariate and multivariate Cox analysis of survival

Parameter	Overall survival					
	Univariate Cox analysis			Multivariate Cox analysis		
	HR	95% CI	*p* value	HR	95% CI	*p* value
Age						
≤ 50 years	**4.172**	**1.673–10.402**	**0.002**	**3.767**	**1.500–9.461**	**0.005**
> 50 years						
Tumour grade						
G1	1.235	0.887–1.719	0.212			
G2						
G3						
Tumour size						
pT1	**1.028**	**1.010–1.045**	**0.002**	**2.012**	**1.469–2.754**	**< 0.0001**
pT2						
pT3						
pT4						
Lymph nodes						
pN0	**1.921**	**1.010–1.045**	**0.038**	**2.294**	**1.295–4.065**	**0.004**
pN1–pN3						
pNx						
pM						
M0	**11.48**	**1.399–90.427**	**0.023**	**7.378**	**0.936–58.136**	**0.058**
M1						
Stage						
I	**1.025**	**1.006–1.045**	**0.010**	**0.512**	**0.374–0.701**	**< 0.0001**
II						
III						
IV						
Oestrogen receptor						
Negative	1.009	0.999–1.019	0.078			
Positive						
Progesterone receptor						
Negative	0.991	0.972–1.010	0.355			
Positive						
HER2						
Negative	1.002	0.983–1.021	0.847			
Positive						
Molecular tumour types						
Triple negative	1.400	0.612–3.200	0.425			
Other types						
GAL3ST1						
Negative (0)	**0.595**	**0.354–0.999**	**0.046**	**0.604**	**0.351–1.038**	**0.068**
Positive (1–12)						
GAL3ST1 chemotherapy subgroup						
Negative (0)	0.659	0.388–1118	0.112			
Positive (1–12)						

Bold values (*p* < 0.05) are statistically significant

*CI* confidence interval, *Gal3ST1* galactosylceramide sulfotransferase, *HR* hazard ratio, *HER2* human epidermal growth factor receptor 2

## Discussion

It is firmly established that neoplastic transformation and tumour progression are almost invariably associated with changes in the expression of surface glycosphingolipids representing ganglio-series, globo-series, lacto-series and neolacto-series [30, 31]. However, there is limited information on the role played by galactosphingolipids, including sulfatides, in human cancer progression [11, 14, 29]. Therefore, the present study was undertaken to evaluate the role of sulfatides in the malignancy-related properties of breast cancer cells. In this respect, our recent findings on the role of GalCer in breast cancer progression are of special interest [14]. We showed that GalCer affects the tumourigenic and metastatic properties of breast cancer cells as an anti-apoptotic molecule. Based on these data, we hypothesised that GalCer facilitates tumour cells to survive in the hostile tumour microenvironment [14, 32]. Since GalCer is a substrate for GAL3ST1 and a precursor molecule for sulfatide, in the present study we analysed the resistance of breast cancer MDA-MB-231 cells with overexpression of sulfatide (MDA.SUL) cells and sulfatide-negative control MDA-MB-231 (MDA.C) cells to doxorubicin-induced and hypoxia-induced apoptosis. We found that MDA.SUL cells were more sensitive to apoptosis than MDA.C cells. Therefore, we quantified the amounts of GalCer in both cell types, and observed that overexpression of GAL3ST1 substantially decreased GalCer levels in breast cancer cells by increased synthesis of sulfatides, which strongly reinforces the importance of GalCer as an anti-apoptotic molecule. This hypothesis was further supported by our “loss-of-function” cellular model. T47D cells with knockout of the *GAL3ST1* demonstrated increased levels of GalCer and increased resistance to doxorubicin-induced and hypoxia-induced apoptosis. Our results suggested that sulfatide may act as a “pro-apoptotic molecule”, therefore an in-vivo study was undertaken to evaluate the role of sulfatide in breast cancer progression. When MDA.SUL and MDA.C cells were subcutaneously injected into athymic nu/nu mice, control MDA-MB-231 cells formed tumours more efficiently than MDA.SUL cells. Furthermore, immunohistochemical staining of tumour specimens revealed that accumulation of sulfatide is associated with significantly higher numbers of apoptotic cells. This correlates with data obtained using MDA-MB-231 cells with high expression of GalCer, which were significantly more tumourigenic than MDA/LUC-shUGT8 cells with decreased expression of UGT8 [14].

In our earlier study [14], we hypothesised that the anti-apoptotic effects of GalCer are associated with increased conversion of ceramide to GalCer, resulting in a decreased intracellular pool of ceramide as one of the key pro-apoptotic molecules [15]. However, the quantification of ceramide in breast cancer cells with high (MDA/LUC cells) and low (MDA/LUC-shUGT8 cells) production of GalCer revealed that both cell types contain essentially the same amounts of ceramide. Therefore, our data suggest that in breast cancer cells, glycosylation of ceramide is not a major mechanism ensuring resistance of cancer cells to drug-induced apoptosis [33]. Instead, it is likely that the key anti-apoptotic molecule in breast cancer cells is GalCer by itself since its conversion to sulfatide is directly associated with highly increased sensitivity to pro-apoptotic agents. Taken together, we propose that an increased level of sulfatide in breast cancer cells is a limiting factor in breast cancer progression. This contradicts other studies showing that sulfatides contribute to the malignant phenotype of cancer cells as ligands for P-selectin supporting their binding to platelets and endothelial cells [10, 12]. Therefore, to address this discrepancy, we evaluated the sulfatides expressed by breast cancer cells as adhesion molecules for endothelial and platelet P-selectin. Shear flow experiments showed for the first time that sulfatides expressed on the surface of tumour cells mediate their rolling on P-selectin-expressing cells, and do not interact with E-selectin-expressing cells. The role of sulfatides in P-selectin-mediated platelet aggregation was studied intensively [10, 34]. In murine colon cancer cells, it was shown that their removal from the cell surface decreases the formation of cancer cell–platelet aggregates, which leads to attenuation of blood-borne experimental metastases [11]. To assess the role of sulfatide in the formation of heterotypic aggregates, MDA.SUL and MDA.C cells were incubated with activated human platelets. Only MDA.SUL cells formed aggregates with P-selectin-expressing platelets. Importantly, when partially ADP-activated platelets were mixed with MDA.SUL cells, elevated expression of P-selectin was observed in comparison to ADP-activated platelets alone. These data are in agreement with studies showing that the treatment of platelets with exogenous sulfatides led to additional platelet aggregation and further P-selectin expression [10]. However, we have shown for the first time that sulfatide-rich tumour cells can mediate the same activating effect. Based on these results we speculate that additional activation of platelets will enhance platelet–tumour cell aggregation contributing to cancer metastasis [35–38].

**Fig. 8:**
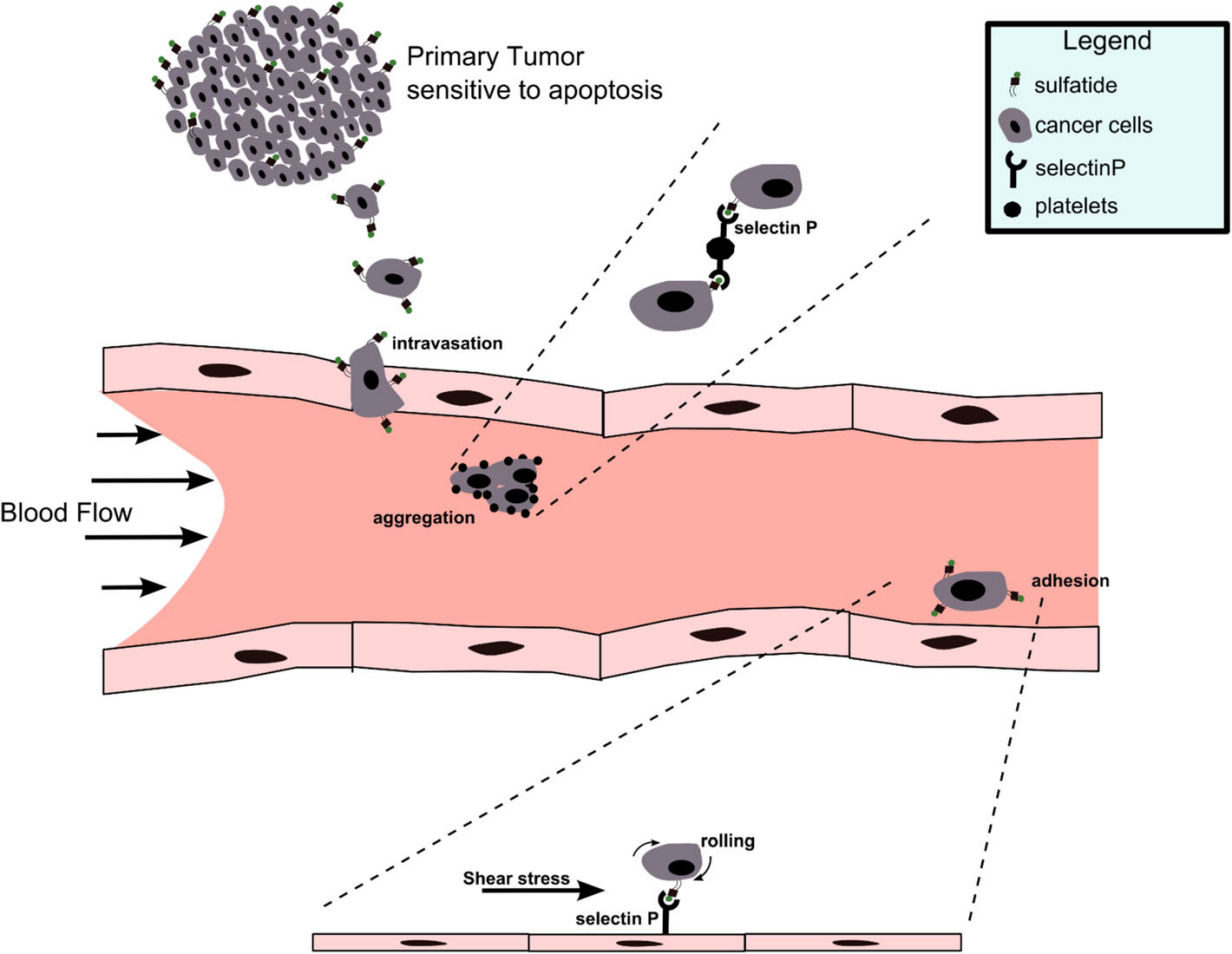
Sulfatides act as a two-edged sword in breast cancer progression

Since earlier studies showed that sulfatides are malignancy-related adhesive molecules [11] and the present findings suggest that sulfatides act as pro-apoptotic molecules, we propose the following hypothesis (Fig. 8). In a primary tumour, cancer cells rich in sulfatides are more prone to apoptosis induced by microenvironmental stressors such as hypoxia, since increased synthesis of this glycolipid decreases the amounts of precursor GalCer, which acts as an anti-apoptotic molecule. However, when such cancer cells escape from the primary tumour and invade lymphatic or blood vessels, sulfatides present on their surface act as adhesive molecules, facilitating cancer cell interactions with platelets and endothelial cells, which in turn increases their metastatic potential. Our proposal based on experimental data is supported by a clinical study with IDC patients. Using IHC, we showed that the expression of GAL3ST1 decreased with tumour malignancy grade with significant differences in GAL3ST1 expression in G1 vs G3 tumours, and that high expression of GAL3ST1 in IDC cells is associated with longer OS of patients. These results suggest that elevated GAL3ST1 levels may be associated with better prognosis.

## Conclusions

We have shown for the first time that sulfatides are not only involved in adhesion of cancer cells, acting as ligands for P-selectin expressed by endothelial cells and platelets, but they also contribute to programmed cell death, acting as “pro-apoptotic molecules”, making cancer cells more prone to environmental stressors such as hypoxia and anticancer drugs such as doxorubicin. Our data strongly suggest that sulfatides are not directly involved in apoptotic processes but changes in their metabolism affect the amounts of GalCer precursor; for example, increased synthesis of sulfatides decreases the amounts of GalCer, which acts directly as an anti-apoptotic molecule [14]. Therefore our findings, which are in full agreement with the hypothesis that the balance between specific sphingolipid species is a critical rheostat for regulation of cellular apoptosis [15], highlight the importance of the sulfatide–galactosylceramide rheostat in cancer progression. Our data also suggest that both molecules can be new targets for breast cancer treatment based on the reversal of tumour cell resistance to microenvironmental-induced and drug-induced apoptosis.

## Additional files

Additional file 1: **Table S1.** Primers used in this study (DOCX 40 kb)

Additional file 2: **Table S2.** Antibodies used in this study (DOCX 31 kb)

Additional file 3: **Figure S1.** (**A**) Expression of GAL3ST1 mRNA in MDA-MB-231, MDA.CTR and MDA.SUL cells. Real-time PCR used to analyse GAL3ST1 mRNA. GAL3ST1 levels normalised against β-actin and MDA-MB-231 cells served as calibrator sample. Results expressed as mean. (**B**) Proliferation of MDA.C and MDA.SUL cells determined using SRB assay. Values shown as mean of six independent replicates. (**C**) Scheme and sequencing results for PCR products of *GAL3ST1* guide RNA (gRNA-bolded) targeting exon 2 in human *GAL3ST1*. Protospacer-adjacent motif (PAM) sequence underlined. Arrows indicate locations of PCR primers (F1: CAGCGTCCTGCTCTCCA, R1: TCACCACCGCAGGAAATC). (**D**) Proliferation of T47D.CRISPR.C and T47D.Δ.GAL3ST1.1 cells determined using SRB assay. Values shown as mean of six independent replicates. (**E**) Western blotting analysis of UGT8 expression in parental T47D cells, control T47D/PURO transduced with pRRL-CMV-IRES-PURO vector alone and T47D/PURO-UGT8 cells transduced with pRRL-CMV-UGT8-IRES-PURO vector containing UGT8 cDNA. (**F**) Immunostaining of neutral glycolipids from T47D, T47D/PURO and T47D/PURO-UGT8 cells separated by HP-TLC with anti-GalCer rabbit polyclonal antibodies (PDF 529 kb)

Additional file 4: **Figure S2.** Binding of sulfatide-expressing MDA.SUL cells to (**A**) P-selectin-expressing CHO-Pro-5 (CHO-Pro/SELP) cells or (**B**) E-selectin-expressing CHO-Pro-5 (CHO-Pro/SELE) cells under flow conditions. CHO cells (dark background) and MDA.SUL cells subjected to fluid shear flow of 1 dyn/cm^2^ and rolling cells shown by numbered arrows. Each image captured every 0.5 s during 5-s perfusion. Corresponding movies added as Additional files 6 and 7 (PDF 506 kb)

Additional file 5: **Video S1.** Binding of sulfatide-expressing MDA.SUL cells to P-selectin-expressing CHO-Pro-5 cells (MP4 290 kb)

Additional file 6: **Video S2.** Binding of sulfatide-expressing MDA.SUL cells to E-selectin-expressing CHO-Pro-5 cells (MP4 383 kb)

Additional file 7: **Figure S3.** Expression of P-selectin on surface of human platelets (10^6^ platelets) activated with ADP at concentration 1 μM (grey) or 5 μM (black). Degree of platelet activation monitored by analysis of P-selectin expression using flow cytometry and monoclonal antibody against P-selectin. Expression level of selectin P in activated platelets determined relative to non-activated platelets (solid line) (PDF 50 kb)

## Abbreviations

Gal3ST1: Galactosylceramide sulfotransferase
GalCer: Galactosyloceramide
HILIC: Hydrophilic interaction liquid chromatography
IDC: Invasive ductal carcinoma
IHC: Immunohistochemistry
IRS: Semi-quantitative immune reactive score
SM4: Sulfatide
SRB: Sulforhodamine B
TLC: Thin-layer chromatography
TUNEL: Terminal transferase dUTP nick end labelling
