# Supplementary material for: Sulfatide decreases the resistance to stress-induced apoptosis and increases P-selectin-mediated adhesion: a two-edged sword in breast cancer progression
Source: Breast Cancer Res. 2018 Nov 6;20:133. doi: 10.1186/s13058-018-1058-z (PMC6219063; doi:10.1186/s13058-018-1058-z)
Supplement: Supplementary file 1 — Table S1. Primers used in this study (DOCX 40 kb) [file 13058_2018_1058_MOESM1_ESM.docx]

| **Table S1. The primers used for PCR amplification and sequence analysis** | | | | |
| --- | --- | --- | --- | --- |
| Primer | Annealing temperature (^o^C) | Primer sequence | Primer length | Reference |
| RT-forGal3ST1 | 52 | 5’ CGCGCTCTTCACTAGTTTCC 3’ | 20 | Newly developed primers |
| RT-revGal3ST1 |  | 5’ CTCGAGTGCAGGTGGAGAG 3’ | 19 |  |
| RT-forUGT | 55 | 5’ CATGGTGTGCCTGTAGTGG 3’ | 19 | Owczarek et al., 2013 |
| RT-revUGT |  | 5’ GAGCCCTCTGACGGTAGC 3’ | 18 |  |
| RT-forAKT | 55 | 5’ ACCACACCTTCTACAATGAGC 3’ | 21 | Suchanski et al., 2017 |
| RT-revAKT |  | 5’ GATAGCACAGCCTGGATAGC 3’ | 20 |  |
| forBgl II-SELP | 50 | 5’ CGG**AGATCT**GCCATGGCCAA 3’  **Bgl II** | 20 | Newly developed primers |
| revBgl II-SELP |  | 5’ CGG**AGATCT**TCAAATGCAGC 3’  **Bgl II** | 20 |  |
| forEcoRI-Gal3ST1 | 48 | 5’ G**GAATTC**CATGCTGCCACCGCAGAA3’  **EcoRI** | 25 | Newly developed primers |
| revMluI-Gal3ST1 |  | 5’ GA**ACGCGT**TCACCACCGCAGGAAAT3'  **Mlu I** | 25 |  |
| F1-exon/Gal3ST1 | 58 | 5’ CAGCGTCCTGCTCTCCA 3’ | 17 | Newly developed primers |
| R1-exon/Gal3ST1 |  | 5’ TCACCACCGCAGGAAATC 3’ | 18 |  |
