# Supplementary material for: Sulfatide decreases the resistance to stress-induced apoptosis and increases P-selectin-mediated adhesion: a two-edged sword in breast cancer progression
Source: Breast Cancer Res. 2018 Nov 6;20:133. doi: 10.1186/s13058-018-1058-z (PMC6219063; doi:10.1186/s13058-018-1058-z)
Supplement: Supplementary file 2 — Table S2. Antibodies used in this study (DOCX 31 kb) [file 13058_2018_1058_MOESM2_ESM.docx]

| **Table S2. List of antibodies** | | | | | |
| --- | --- | --- | --- | --- | --- |
| **Antibody** | **Clone** | **Host** | **Source** | **Application** | **Dilution** |
| CD62P/P-selectin | CTB201 | mouse | Santa Cruze Biotechnology | Flow cytometry | FACS (1:50) |
| [Anty-rabbit FITC](http://www.abcam.com/goat-rabbit-igg-hl-fitc-ab97050.html) | polyclonal | goat | Invitrogen | Flow cytometry | FACS (1:200) |
| GAL3ST1 | Polyclonal | rabbit | Novus Biologicals | Immunohistochemistry | IHC (1:100) |
| GAL3ST1 | polyclonal | rabbit | Pierce | Western blotting | WB (1:1000) |
| UGT8 | polyclonal | rabbit | US-Biological | Western blotting | WB (1:1000) |
| AKT | mAbcam 8226 | mouse | Abcam | Western blotting | WB (1:2000) |
| sulfatide | O4 | mouse | R&D | TLC binding assay | TLC (1:100) |
| GalCer | polyclonal | rabbit | Sigma | TLC binding assay | TLC (1:200) |
| Anti- mouse HRP | polyclonal | goat | Dako | Western blotting | WB (1:5000) |
| Anti- rabbit HRP | polyclonal | goat | Dako | Western blotting | WB (1:2000) |
