# Supplementary figures and images for: Sulfatide decreases the resistance to stress-induced apoptosis and increases P-selectin-mediated adhesion: a two-edged sword in breast cancer progression
Source: Breast Cancer Res. 2018 Nov 6;20:133. doi: 10.1186/s13058-018-1058-z (PMC6219063; doi:10.1186/s13058-018-1058-z)

A

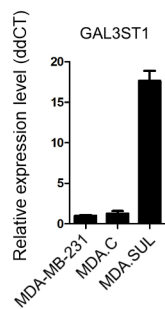

B

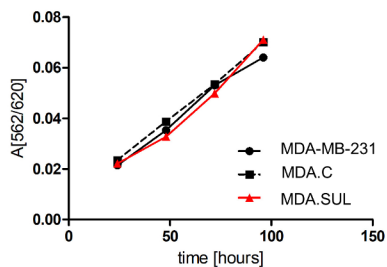

C

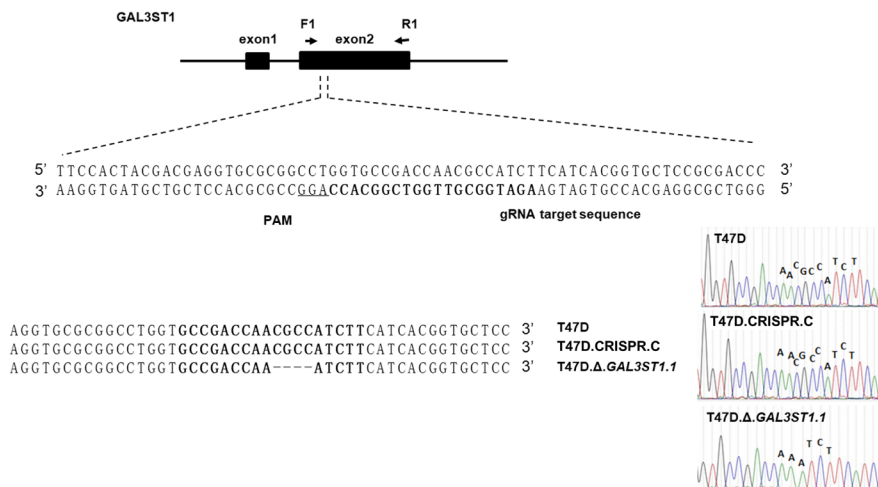

D

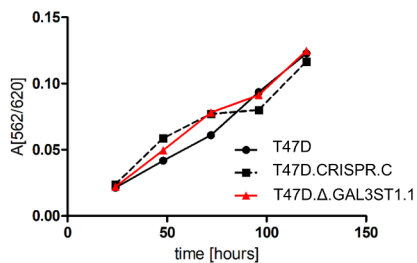

E

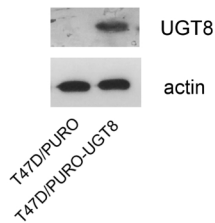

F

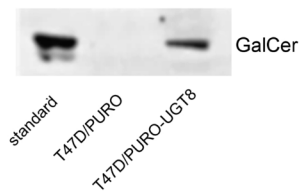

Supplement: Supplementary file 3 — Figure S1. (A) Expression of GAL3ST1 mRNA in MDA-MB-231, MDA.CTR and MDA.SUL cells. Real-time PCR used to analyse GAL3ST1 mRNA. GAL3ST1 levels normalised against β-actin and MDA-MB-231 cells served as calibrator sample. Results expressed as mean. (B) Proliferation of MDA.C and MDA.SUL cells determined using SRB assay. Values shown as mean of six independent replicates. (C) Scheme and sequencing results for PCR products of GAL3ST1 guide RNA (gRNA-bolded) targeting exon 2 in human GAL3ST1. Protospacer-adjacent motif (PAM) sequence underlined. Arrows indicate locations of PCR primers (F1: CAGCGTCCTGCTCTCCA, R1: TCACCACCGCAGGAAATC). (D) Proliferation of T47D.CRISPR.C and T47D.Δ.GAL3ST1.1 cells determined using SRB assay. Values shown as mean of six independent replicates. (E) Western blotting analysis of UGT8 expression in parental T47D cells, control T47D/PURO transduced with pRRL-CMV-IRES-PURO vector alone and T47D/PURO-UGT8 cells transduced with pRRL-CMV-UGT8-IRES-PURO vector containing UGT8 cDNA. (F) Immunostaining of neutral glycolipids from T47D, T47D/PURO and T47D/PURO-UGT8 cells separated by HP-TLC with anti-GalCer rabbit polyclonal antibodies (PDF 529 kb) [file 13058_2018_1058_MOESM3_ESM.pdf]

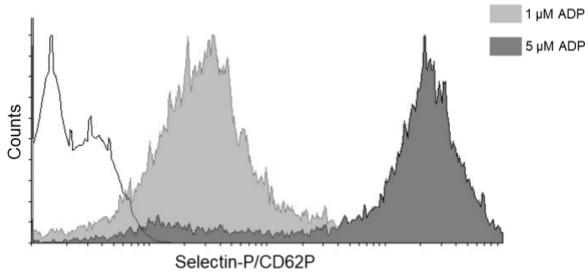

Supplement: Supplementary file 7 — Figure S3. Expression of P-selectin on surface of human platelets (106 platelets) activated with ADP at concentration 1 μM (grey) or 5 μM (black). Degree of platelet activation monitored by analysis of P-selectin expression using flow cytometry and monoclonal antibody against P-selectin. Expression level of selectin P in activated platelets determined relative to non-activated platelets (solid line) (PDF 50 kb) [file 13058_2018_1058_MOESM7_ESM.pdf]
