# Supplementary material for: Sulfatide decreases the resistance to stress-induced apoptosis and increases P-selectin-mediated adhesion: a two-edged sword in breast cancer progression
Source: Breast Cancer Res. 2018 Nov 6;20:133. doi: 10.1186/s13058-018-1058-z (PMC6219063; doi:10.1186/s13058-018-1058-z)

A Binding of MDA.SUL cells to CHO-Pro5/SELP cells expressing selectin P

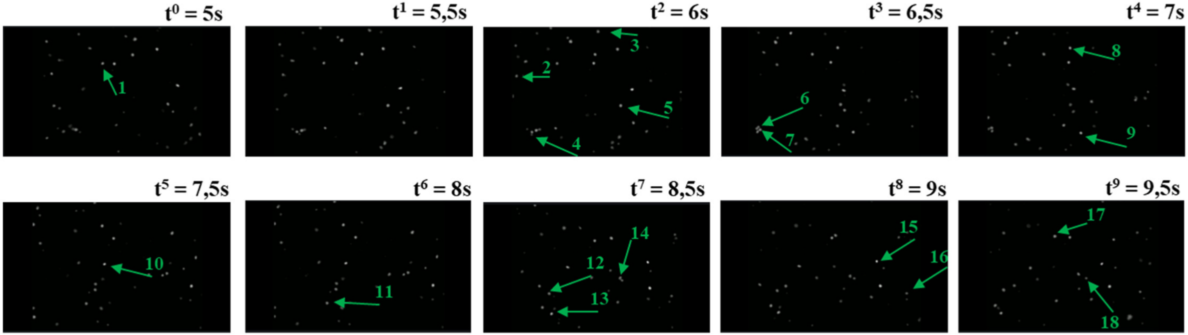

B Binding of MDA.SUL cells to CHO-Pro5/ELAM cells expressing selectin E

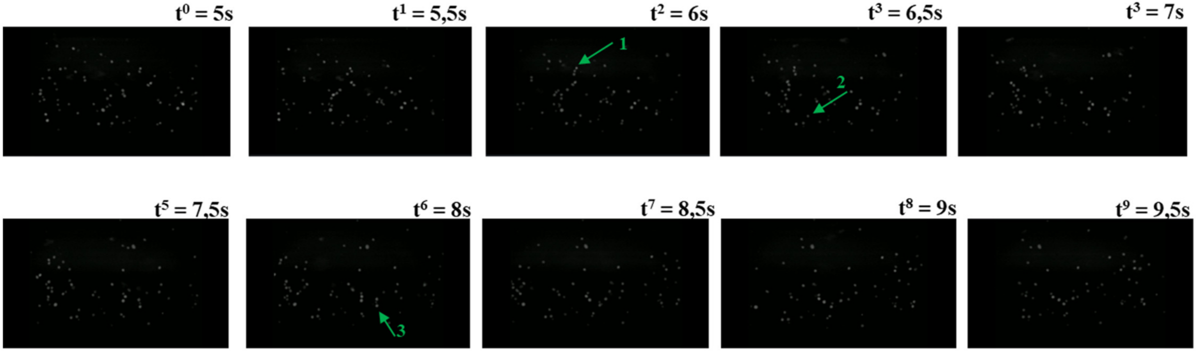

Supplement: Supplementary file 4 — Figure S2. Binding of sulfatide-expressing MDA.SUL cells to (A) P-selectin-expressing CHO-Pro-5 (CHO-Pro/SELP) cells or (B) E-selectin-expressing CHO-Pro-5 (CHO-Pro/SELE) cells under flow conditions. CHO cells (dark background) and MDA.SUL cells subjected to fluid shear flow of 1 dyn/cm2 and rolling cells shown by numbered arrows. Each image captured every 0.5 s during 5-s perfusion. Corresponding movies added as Additional files 6 and 7 (PDF 506 kb) [file 13058_2018_1058_MOESM4_ESM.pdf]
